# Primary Immunodeficiency Disorders in India—A Situational Review

**DOI:** 10.3389/fimmu.2017.00714

**Published:** 2017-06-19

**Authors:** Ankur Kumar Jindal, Rakesh Kumar Pilania, Amit Rawat, Surjit Singh

**Affiliations:** ^1^Allergy Immunology Unit, Advanced Pediatrics Centre, Postgraduate Institute of Medical Education and Research, Chandigarh, India

**Keywords:** primary immunodeficiencies, intravenous immunoglobulin, hematopoietic stem cell transplantation, India, newborn screening

## Abstract

Primary immunodeficiency disorders (PIDs) are a group of genetic defects characterized by abnormalities of one or more components of the immune system. While there have been several advances in diagnosis, management, and research in the field of PIDs, they continue to remain underdiagnosed, especially in the less affluent countries. Despite several limitations and challenges, India has advanced significantly in the field of PIDs in the last few years. In this review, we highlight the progress in the field of PIDs in India over the last 25 years, the difficulties faced by clinicians across the country, the current state of PIDs in India and the future prospects.

## Introduction

Primary immunodeficiency disorders (PIDs) are a group of genetic defects characterized by abnormalities of one or more components of the immune system. More than 300 genetically defined single-gene inborn errors of immunity are now recognized as a cause of PID (1). The International Union of Immunological Societies Expert Committee on classification of PIDs has recently updated the phenotypic classification for PIDs (2). These diseases may affect innate immunity and/or adaptive immunity, and they result in a wide range of manifestations including, but not limited to, susceptibility to infections, autoimmunity, inflammation, allergy, and malignancy. Contrary to common perception PIDs are not uncommon and may have prevalence rates as high as 1:1,200 in the general population (3). The diagnosis of PIDs is often delayed, or even missed altogether, especially in areas where infectious diseases are common as is the case in most developing countries.

While there have been several advances in diagnosis, management, and research in the field of PIDs, they continue to remain underdiagnosed, especially in the less affluent countries. This is largely because of lack of awareness about these conditions both among the laity and medical professionals.

Although there are no nationwide data on prevalence of PIDs in India, based on statistical projections it is estimated that the number of patients with PID is likely to be more than one million (4, 5). Hitherto, there was also paucity of published literature about PIDs from India as very few centers in the country had the clinical experience, laboratory facilities, and technical wherewithal for diagnosis and management of these conditions. As a result, an overwhelming majority of these patients continued to remain undiagnosed and untreated.

However, despite these limitations and challenges, India has advanced significantly in the field of PIDs in the last few years. The Indian Society for Primary Immune Deficiency (ISPID) was founded in 2011 in close liaison with the Foundation for Primary Immunodeficiency Diseases (FPID), USA. The Indian Council of Medical Research (ICMR) has also been at the forefront of activities related to PIDs in India. In this review, we highlight the progress in the field of PIDs in India over the last 25 years, the difficulties faced by clinicians across the country, the current state of PIDs in India and the future prospects.

## Status of PIDs in India Prior to 2011

The first two cases of PIDs in India were reported in the year 1964—these were two case reports of boys with Wiskott–Aldrich syndrome (WAS) and agammaglobulinemia, respectively (6, 7). Subsequently, five families with ataxia telangiectasia were reported in the year 1973 (8). Since then several cases of PIDs have been reported from Postgraduate Institute of Medical Education and Research (PGIMER), Chandigarh and many other institutions across the country; however, the diagnosis was largely clinical and the immunological workup rather limited. Prior to 1992 (between 1975 and 1991), 59 cases of PIDs had been diagnosed in the country (5). Gupta et al. published their experience of the spectrum of PIDs seen at two large pediatric centers in the country [Advanced Pediatrics Centre, PGIMER, Chandigarh and the National Institute of Immunohaematology (NIIH) and B.J. Wadia Children’s Hospital, Mumbai] and highlighted the difficulties in identification, evaluation (including molecular diagnosis) and management of these patients (5). The data were collected from 1992 onward. Between 1992 and 2010, a total of 153 and 122 patients had been diagnosed to have PIDs at Chandigarh and Mumbai respectively. There were important differences in spectrum of PIDs seen at both these centers. Antibody deficiency disorders [including X-linked agammaglobulinemia (XLA), common variable immunodeficiency (CVID), selective IgA deficiency with or without IgG2 subclass deficiency, isolated IgG2 subclass deficiency, and transient hypogammaglobulinemia of infancy] were the most common PIDs diagnosed in Chandigarh; however, the most common PID diagnosed at Mumbai was familial hemophagocytic lymphohistiocytosis (HLH). Cases of disorders of immune regulation (including HLH and autoimmune lymphoproliferative syndrome) were predominantly seen at Mumbai while only a few cases had been diagnosed at Chandigarh. Similarly, phagocyte defects [including neutropenia, leukocyte adhesion defect (LAD), and disorders of IFNγ-IL12 pathway] were predominantly being diagnosed at Mumbai with only a handful of cases at Chandigarh. On the other hand, WAS, hyper-IgE syndrome, ataxia telangiectasia, hereditary angioedema, and chronic mucocutaneous candidiasis were more common at Chandigarh. There could be several reasons for the difference in spectrum of PID seen at these two centers. These could be (1) difference in genetic constitution of the population in North and West India due to differences in racial ethnicities; (2) referral bias (the Mumbai centre is primarily a hemato-oncology unit and tends to attract more cases of HLH and phagocytic defects; the Chandigarh facility on the other hand is a pediatric center and sees the entire spectrum of PIDs); and (3) difference in rate of consanguineous marriages (much higher in Western and Southern India as compared to North India). The diagnosis of PIDs at both centers was being established based on clinical manifestations supplemented by few laboratory investigations including protein expression by flow cytometry and a few functional assays. Facilities for molecular analysis were limited at that time. However, both centers had been able to get some molecular analyses done on individual patients for diagnostic purposes through collaborative efforts with overseas laboratories specializing in PIDs. Major challenges in the diagnosis and management of cases of PID in India prior to 2011 were lack of awareness among general population and physicians, lack of appropriate diagnostic facilities and cost constraints in management of patients diagnosed to have PIDs especially with regard to access to immunoglobulin replacement and hematopoietic stem cell transplantation (HSCT).

### The FPID^1^

The FPID was co-founded by Dr. Sudhir Gupta and Dr. Abha Gupta in the USA. This foundation has the avowed aim of supporting education, early diagnosis, genetic counseling, therapy, and research in PID in both India and the USA. The FPID is spearheading efforts at establishing PID centers in India and is currently supporting activities at five institutions in the country, i.e., at PGIMER, Chandigarh; Sir Ganga Ram Hospital (SGRH), New Delhi; Sanjay Gandhi Postgraduate Institute of Medical Sciences (SGPGIMS), Lucknow; Nizam’s Institute of Medical Sciences (NIMS), Hyderabad; and NIIH, Mumbai (Figure S1 in Supplementary Material). It is also supporting treatment of some patients with hypogammaglobulinemia in India by provision of intravenous immunoglobulin (IVIg) replacement and partial support toward HSCT.

### Indian Society for Primary Immune Deficiency^2^

The ISPID was registered in March 2011. Very few developing countries have a dedicated society for PID—India is one of them. ISPID has been working toward increasing awareness regarding PIDs and establishment of diagnostic support and research centers in the country. ISPID organizes a national and an international conference on PID every alternate year and a Continuing Medical Education program every year with the support of FPID, USA. Till date four such national and international conferences have been organized by the ISPID in India. The faculty at these conferences has consisted of world renowned scientists at the cutting edge of research in PID.

### Indian Patients Society for Primary Immunodeficiency (IPSPI)^3^

Indian Patients Society for Primary Immunodeficiency is a national non-profit organization for PIDs, which was established in 2004 and registered in 2005. It was the first patient society for PIDs in our country. It is based at Bhubaneshwar in Eastern India. IPSPI is affiliated with the International Patient Organization for Primary Immunodeficiencies. This organization works to improve the quality of life of individuals with PIDs in India.

### Primary Immunodeficiency Patients Welfare Society (PIDPWS)^4^

The second patient care society for PID in India was founded in April 2012 and registered in September 2012. Like the IPSPI, this society has also been established by parents of the children suffering from PIDs. PIDPWS is based at Bengaluru in South India and has been working for the improvement in quality of life of PID patients.

## Status of PIDs in India after 2011

Since the inception of ISPID in 2011, there has been a significant progress in the spread of awareness and setting up of diagnostic facilities for PIDs. The PGIMER, Chandigarh initiated a 3 years postdoctoral (DM) training course in Pediatric Clinical Immunology and Rheumatology in January 2014. This course is the first of its kind in India. The basic aim of this program is to train pediatric fellows and to provide them enough expertise in the field of immunology that can be disseminated across the country. Two fellows have already completed their training and are now working in Guwahati (East India) and Bengaluru (South India), respectively. In addition, six more trainees are currently pursuing this course at the institute. The institute has also started a 3 years postdoctoral (DM) training course for adult physicians in July 2014. Though several other institutes in the country have been running similar postdoctoral (DM) courses in Clinical Immunology (e.g., SGPGIMS, Lucknow and Jawaharlal Institute of Postgraduate Institute of Medical Education and Research, Puducherry) and in Rheumatology (e.g., Madras Medical College, Chennai; NIMS, Hyderabad; Christian Medical College, Vellore; and King George’s Medical University, Lucknow). However, the major focus of these institutes has been clinical rheumatology rather than PIDs.

## Centre for Advanced Research (CAR) for PIDs in India

Clearly, PID care in India is still in its infancy, and we have a long way to go. There are very few centers in the country with clinical expertise and diagnostic facilities for PIDs. As a result, majority of patients with PIDs continue to remain undiagnosed. The ICMR helped set up the CAR in primary immunodeficiency disorders at PGIMER, Chandigarh in January 2015. The second such ICMR CAR in PID has recently been set up at the NIIH, Mumbai. The major objectives for these CARs are training of physicians/scientists, development and standardization of diagnostic facilities, establishment of normograms, and creation of state of the art facilities for diagnosis and management of PIDs.

### Asia Pacific Society for Immunodeficiencies (APSID)^5^

Asia Pacific Society for Immunodeficiencies was established in April 2015 by a group of Asian pediatricians and immunologists working in the field of PIDs. The aim of this society is to improve management of PIDs through increasing awareness and promoting collaboration among member countries. The APSID has been organizing Spring Schools for young clinicians who are in training for immunology and infectious diseases. The first conference of the APSID was held in 2016 in Hong Kong from 30th April to 1st May 2016. The APSID would provide a much needed fillip to PID-related activities across countries in the large Asia Pacific region which is home to ~60% of the world population.

## Publications on PID from India—A Major Thrust in Last 6 Years

There has been a significant increase in the number and quality of publications on PID from India in the last 6 years. Publications prior to 2011 were mostly case reports and clinical reviews; however, the more recent ones contain a plethora of molecular and genetic details. This is a welcome change.

### Search Strategy

The following search terms were used to find out the number of PubMed publications in PID in the last 50 years in India: India and Primary immune deficiency; Wiskott–Aldrich syndrome and India; Severe combined immunodeficiency and India; X-linked agammaglobulinemia and India; Chronic granulomatous disease and India; Neutropenia and India; Leucocyte adhesion defect and India; Hyper IgE syndrome and India; Hyper-IgM syndrome and India; Chédiak–Higashi syndrome and India; Hemophagocytic lymphohistiocytosis and India; DOCK-8 deficiency and India; Papillon–Lefevre syndrome and India; Ataxia telangiectasia and India; Mendelian susceptibility to mycobacterial diseases and India; Chronic mucocutaneous candidiasis and India; HSCT and Primary Immune deficiency and India; Intravenous immunoglobulin and India and Primary immunodeficiency.

**Figure 1:**
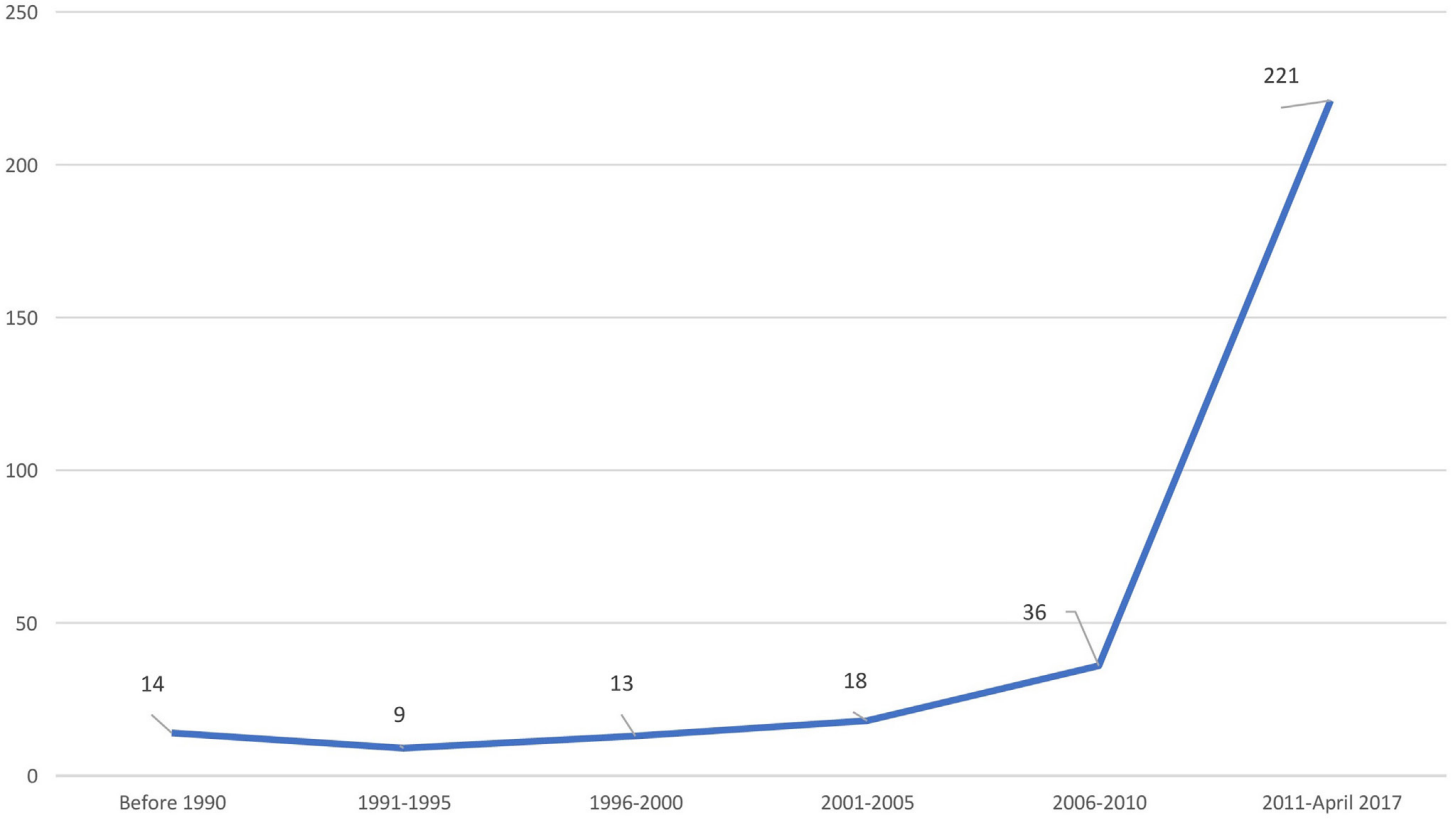
Publications on PIDs from India.

Based on this search strategy, we were able to access 311 publications on PID from India (Figure 1).

## Immunoglobulin Replacement Therapy in India

Immunoglobulin replacement therapy is the cornerstone of treatment for antibody deficiency diseases, arguably the most common clinically significant group of PIDs. Immunoglobulin can be administered by both intravenous and subcutaneous routes; however, subcutaneous immunoglobulin preparations are presently not being marketed in India. Cost of IVIg therapy is a major constraint in the management of children with PIDs in India, as also in other developing countries. However, with recent policy changes initiated by the Federal Government there has been a significant price reduction in IVIg formulations in India. A 5 g vial of IVIg presently costs around 120–140 USD. With the help of philanthropist organizations, FPID and several state governments, IVIg therapy is now being offered to many patients with hypogammaglobulinemia in India. The state government of Punjab (in North India) has taken a major step in this context and is freely providing IVIg to all patients with hypogammaglobulinemia. The state government of Karnataka (in South India) has also taken a major policy initiative and has agreed, in principle, to provide IVIg to patients with PIDs. Because of cost constraints, several of our patients with hypogammaglobulinemia are unable to get the recommended dose of IVIg. Suri et al. recently analyzed the trough levels of IgG in a cohort of 14 boys with XLA at a single center in Chandigarh and correlated the levels with the monthly replacement dose of IVIg and risk of infections (9). This study revealed that the mean dose of IVIg was 414 mg/kg/month and the mean trough IgG level of the entire cohort was 435 mg/dl. It was further observed that a median dose of IVIg of 397 mg/kg and a median trough IgG level of 354 mg/dl was protective for majority of the children. These cutoff levels are much lower that what has hitherto been considered the norm in patients with hypogammaglobulinemia. However, these findings need to be corroborated by clinical results on long-term follow-up.

## Hematopoietic Stem Cell Transplantation for PIDs

Management of children with PIDs requires a multidisciplinary approach and HSCT plays a major role in improving the outcome of patients with PID. HSCT provides a definitive cure for several primary immune deficiencies including severe combined immunodeficiency (SCID), WAS, chronic granulomatous disease (CGD), HLH, and many other immunodeficiency disorders (10). There are little published data regarding the outcome of HSCT for PID from India (11). The major hurdles so far have been the lack of awareness about diagnosis and availability of expertise for carrying out this procedure in the setting of PID. Even though many centers in India are now routinely performing HSCT for a variety of malignant diseases in both children and adults, there is only a minority that have the requisite expertise for carrying out transplant for PIDs (Figure S1 in Supplementary Material). Kapoor and Raj have recently published the data on HSCT in PID from India (11). Approximately 100 transplants have so far been carried out in 10 centers across the country. The indications included SCID, HLH and WAS.

## Vaccines in PID

Children with PIDs may not mount an immune response to a given vaccine (e.g., response to pneumococcal vaccine in children with XLA), and these vaccines may not be indicated (12). On the other hand, a live vaccine may be deleterious as once a live bacterium or virus from a vaccine strain is administered to these patients, it is very difficult to eradicate this organism from the body of an immunocompromised child (12). Oral live polio vaccine is still being used in India as a part of Polio Eradication and Endgame Strategic Plan and majority of children receive the first dose at birth. Subsequently, about three to four doses are given in the first year of life either as part of routine immunization or pulse polio immunization program (which is conducted on two designated days per year, and all children below 5 years are given a dose of oral polio on these days). Similarly, all babies born in India receive BCG vaccination at birth. This contrasts with the policy in some of the developed countries where BCG is either not administered at all or, if administered, it is deferred until results of T-cell receptor excision circle (TREC) assay are available. Similarly, OPV has been discontinued in most of the developed countries. Thus, babies born in India with a significant cell mediated immune defect are at risk of developing disseminated BCG infection as well as vaccine-associated paralytic poliomyelitis (VAPP) (4). Recently, a case was reported from India in whom VAPP caused by vaccine-derived poliovirus (VDPV) (strain type 2) was the first presenting manifestation of CVID (13). The recent update on VDPV from India (January 2015–May 2016) revealed three cases of VDPV (one each in XLA, CVID, and SCID) (14). The Jeffrey Modell Foundation in collaboration with Centers for Disease Control and Prevention, WHO—Global Polio Laboratory Network and The Task Force for Global Health recently conducted a surveillance to determine the prevalence of poliovirus excretion in immunodeficient children across 13 different countries (15). Twenty-three children with XLA from Chandigarh, India, were recruited in this multicenter study, and stool samples were cultured for detection of poliovirus excretion. While none of the patients from Chandigarh were excreting poliovirus, 13 patients from Iran, Tunisia, Turkey, and the Russian Federation were found to be doing so. These findings have implications for health planners and vaccinologists all over the world.

## Newborn Screening (NBS) in PID

Early diagnosis of PIDs is of paramount importance before these children acquire a significant infection (16). HSCT is a curative treatment for several PIDs and the best outcome can be achieved if the transplant is performed before these children acquire an infection (17). SCID is usually not apparent at birth and these children can only be detected by NBS before they get any major illness. NBS for SCID is usually done using TREC assay, which is a measure of thymic output of T cells and is a sensitive assay (18, 19). NBS for SCID is now being routinely carried in all states of USA and many other developed countries. However, the cost of TREC assay is a major constraint for its inclusion in the NBS program in India. In the current scenario, where routine NBS is not available even for more common disorders such as hypothyroidism, NBS for SCID in the near future appears far-fetched. Early screening for SCID will also prevent administration of BCG and other live vaccines to these patients. The FPID is actively considering pilot studies on NBS for SCID in India.

## Non-Infectious Complications in PID

Patients with PID are also predisposed to develop autoimmune, inflammatory, and allergic complications. While children with CGD have significant inflammatory disorders (20), autoimmune complications are characteristic of CVID and WAS. PIDs can also be associated with several malignancies. We recently reported a case of WAS with Hodgkin lymphoma from Chandigarh (21). A case of diffuse large B-cell lymphoma was reported in another child with WAS from Vellore in 2014 (22) and a case of multifocal extranodal non-Hodgkin lymphoma in a child with combined immunodeficiency and Epstein–Barr virus infection (23). We have also seen leiomyoma of liver in a boy with ataxia telangiectasia (unpublished data).

## Status and Prospects of PID India

**Figure 2:**
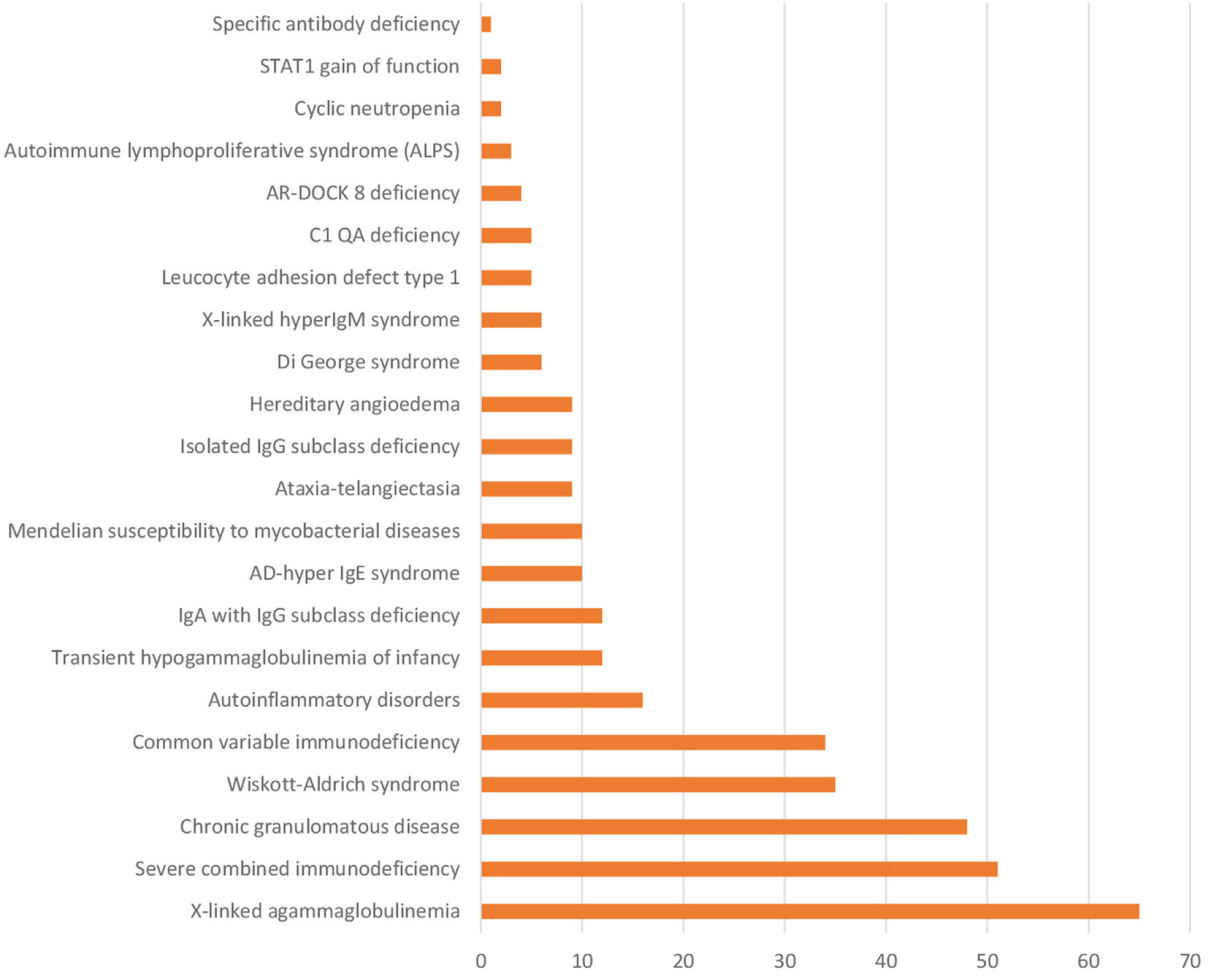
Number of patients with various PIDs at Postgraduate Institute of Medical Education and Research, Chandigarh (January 1990–March 2017).

The spectrum of PIDs diagnosed at a single center in India (PGIMER, Chandigarh) is shown in Figure 2. It is apparent that this spectrum is perhaps no different from that in any other developed country. In addition, PGIMER and many other institutions in India have now developed the facility for genetic analysis for common immunodeficiency diseases. At Chandigarh, facilities for genetic testing for WAS, CGD, XLA, HLH (PRF-1), and X-linked hyper-IgM syndrome are in place. Similarly, genetic testing for HLH, LAD, and several other PIDs is available at NIIH, Mumbai. In addition, a prenatal screening program is being offered to patients with PIDs at PGIMER, Chandigarh as also at NIIH, Mumbai and SGRH, New Delhi (Figure S1 in Supplementary Material). This has been a major advance in the field of PIDs in India. An effort is being made to establish a PID registry for the country; however, it has not been established till date.

## Ethnic Constitution and Consanguinity in India

India has large variation in ethnicity across various geographic regions with six different major religions being followed in the country (24). There is a huge burden of consanguineous and endogamous marriages in India (25, 26). The figure varies from 1% in some states to as high as 38% in others. The rate of consanguineous marriages is much higher in southern parts of India (e.g., 38% in Tamil Nadu, 29% in Andhra Pradesh, and 28% in Karnataka) as compared to northern states (27). Several autosomal recessive disorders (as for instance thalassemia) are more commonly reported from regions in India that have increased rate of consanguineous and endogamous marriages, data on PIDs are scare. Our own data from PGIMER, Chandigarh have shown that proportion of autosomal recessive CGD is higher than the X-linked CGD and this is likely due to the high incidence of endogamous and consanguineous marriages in this part of the world (28). However, the increased likelihood of finding an HLA-matched donor in immediate and extended families appears to be the “silver lining” amidst the baneful effects of consanguinity and endogamy (29, 30).

In conclusion, the outcome of patients with PIDs in India appears to be brighter with an increased awareness of these disorders among physicians and specialists. Availability of diagnostic facilities including candidate gene sequencing and prenatal diagnosis for common PIDs in few centers is also a big step forward from the last decade. HSCT is also being performed at more centers now. Although progress has been made during the last few years, we still have a long arduous journey ahead to provide PID patients in India the standard of care that they rightfully deserve.

## Author Contributions

AJ and RP—initial draft of manuscript, editing of manuscript, revision of manuscript, and data collection. AR and SS—editing and critical revision of manuscript, data collection, and final approval of manuscript.

## Conflict of Interest Statement

The authors declare that the research was conducted in the absence of any commercial or financial relationships that could be construed as a potential conflict of interest.

## Footnotes

^1^www.fpid.org.

^2^www.ispid.org.in.

^3^www.ipspiindia.org.

^4^www.pidindia.net.

^5^www.paed.hku.hk.

## Supplementary Material

Figure S1 Geographical map of India depicting PID centers across the country with different levels of care and facilities.

## Abbreviations

ALPS, autoimmune lymphoproliferative syndrome; APSID, Asia Pacific Society for Immunodeficiencies; CAR, Centre for Advanced Research; CDC, Centers for Disease Control and Prevention; CGD, chronic granulomatous disease; CMC, Christian Medical College; CME, Continued Medical Education; CVID, common variable immunodeficiency; FPID, Foundation for Primary Immunodeficiency Diseases; HLH, hemophagocytic lymphohistiocytosis; HSCT, hematopoietic stem cell transplantation; ICMR, Indian Council of Medical Research; IPOPI, International Patient Organization for Primary Immunodeficiencies; IPSPI, Indian Patients Society for Primary Immunodeficiency; ISPID, Indian Society for Primary Immune Deficiency; IUIS, International Union of Immunological Societies; IVIg, intravenous immunoglobulin; JIPMER, Jawaharlal Institute of Postgraduate Institute of Medical Education and Research; JMF, Jeffrey Modell Foundation; KGMU, King George’s Medical University; LAD, leukocyte adhesion defect; MMC, Madras Medical College; NBS, newborn screening; NIIH, National Institute of Immunohaematology; NIMS, Nizam’s Institute of Medical Sciences; PGIMER, Postgraduate Institute of Medical Education and Research; PID, Primary Immunodeficiency disorder; PIDPWS, Primary Immunodeficiency Patients Welfare Society; SCID, severe combined immunodeficiency; SGPGIMS, Sanjay Gandhi Postgraduate Institute of Medical Sciences; SGRH, Sir Ganga Ram Hospital; TREC, T-cell receptor excision circle; VAPP, vaccine-associated paralytic poliomyelitis; VDPV, vaccine-derived poliovirus; WAS, Wiskott–Aldrich syndrome; XLA, X-linked agammaglobulinemia.
