# Supplementary material for: Primary Immunodeficiency Disorders in India—A Situational Review
Source: Front Immunol. 2017 Jun 19;8:714. doi: 10.3389/fimmu.2017.00714 (PMC5474457; doi:10.3389/fimmu.2017.00714)
Supplement: Figure S1 — Geographical map of India depicting PID centers across the country with different levels of care and facilities. [file Presentation_1.PPTX]

## Slide 1
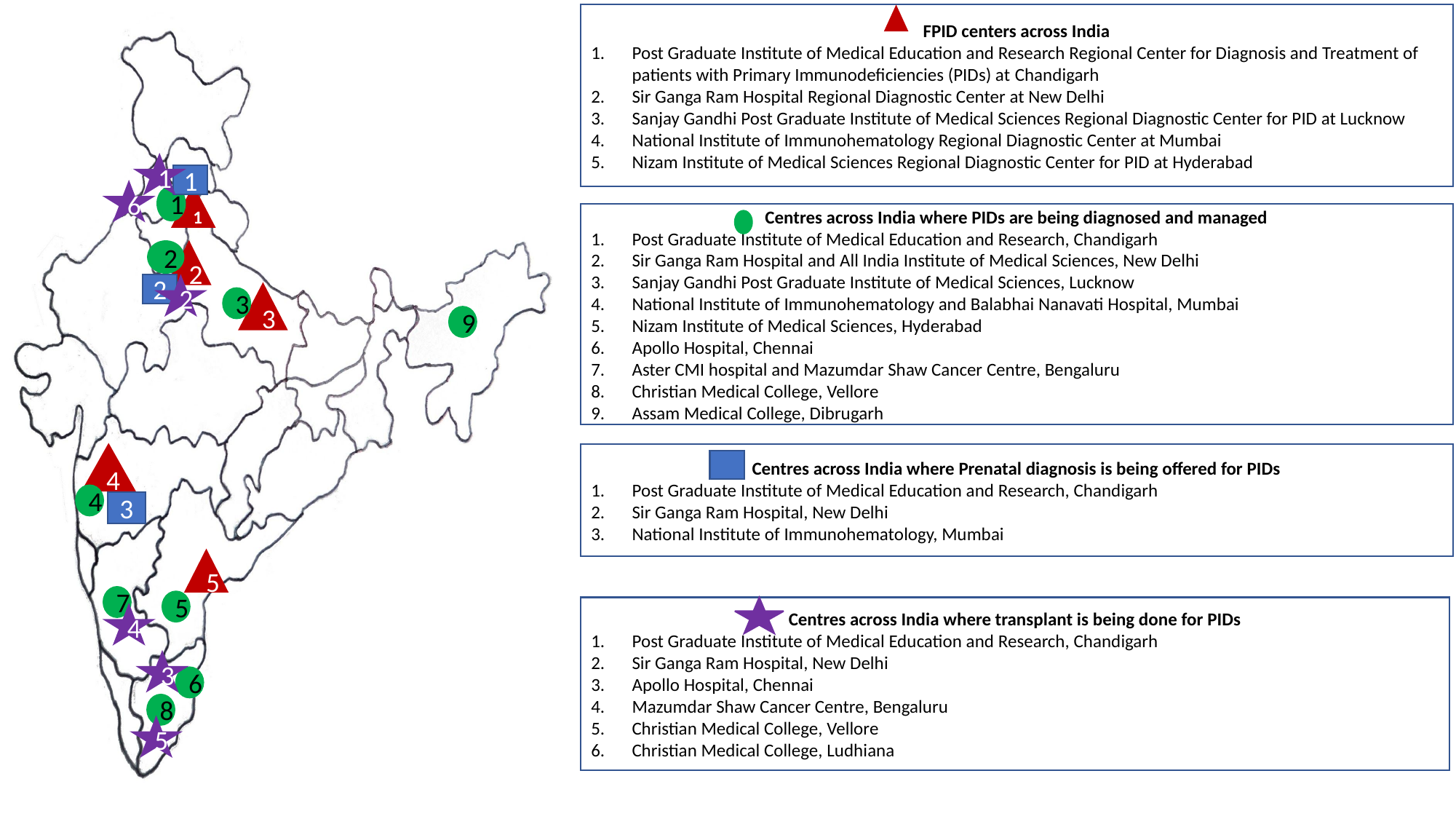

FPID centers across India
Post Graduate Institute of Medical Education and Research Regional Center for Diagnosis and Treatment of patients with Primary Immunodeficiencies (PIDs) at Chandigarh
Sir Ganga Ram Hospital Regional Diagnostic Center at New Delhi
Sanjay Gandhi Post Graduate Institute of Medical Sciences Regional Diagnostic Center for PID at Lucknow
National Institute of Immunohematology Regional Diagnostic Center at Mumbai
Nizam Institute of Medical Sciences Regional Diagnostic Center for PID at Hyderabad
1
2
3
4
5
1
1
6
1
Centres across India where PIDs are being diagnosed and managed
Post Graduate Institute of Medical Education and Research, Chandigarh
Sir Ganga Ram Hospital and All India Institute of Medical Sciences, New Delhi
Sanjay Gandhi Post Graduate Institute of Medical Sciences, Lucknow
National Institute of Immunohematology and Balabhai Nanavati Hospital, Mumbai
Nizam Institute of Medical Sciences, Hyderabad
Apollo Hospital, Chennai
Aster CMI hospital and Mazumdar Shaw Cancer Centre, Bengaluru
Christian Medical College, Vellore
Assam Medical College, Dibrugarh
2
2
2
3
9
Centres across India where Prenatal diagnosis is being offered for PIDs
Post Graduate Institute of Medical Education and Research, Chandigarh
Sir Ganga Ram Hospital, New Delhi
National Institute of Immunohematology, Mumbai
4
3
7
5
Centres across India where transplant is being done for PIDs
Post Graduate Institute of Medical Education and Research, Chandigarh
Sir Ganga Ram Hospital, New Delhi
Apollo Hospital, Chennai
Mazumdar Shaw Cancer Centre, Bengaluru
Christian Medical College, Vellore
Christian Medical College, Ludhiana
4
3
6
8
5
